# Excessive Daytime Sleepiness in Depression and Obstructive Sleep Apnea: More Than Just an Overlapping Symptom

**DOI:** 10.3389/fpsyt.2021.710435

**Published:** 2021-09-09

**Authors:** Danwei Zhang, Zhen Zhang, Huihua Li, Kaimo Ding

**Affiliations:** ^1^Department of Psychology, Zhenjiang Mental Health Center, Zhenjiang, China; ^2^Department of Psychiatry, Zhenjiang Mental Health Center, Zhenjiang, China

**Keywords:** excessive daytime sleepiness, depression, obstructive sleep apnea, continuous positive airway pressure, overlapping symptoms

## Abstract

Excessive daytime sleepiness (EDS) is a significant public health concern, with obstructive sleep apnea (OSA) being a common cause, and a particular relationship exists with the severity of depression. A literature search on OSA, depression, and EDS was performed in PubMed. The chosen evidence was limited to human studies. Available evidence was systematically reviewed to ascertain the association of EDS with depression and OSA according to the general population and some specific population subgroups. In addition, effectiveness of continuous positive airway pressure (CPAP) was analyzed as a standard therapy for improving EDS and depression in patients with OSA. In the general population, patients with OSA, and some other subpopulations, the review contributed to: (1) delineating the prevalence of EDS; (2) substantiating the relationship of EDS and depression; (3) presenting the relationship between EDS and OSA; and (4) revealing that the duration of CPAP is crucial for its therapeutic effects in improving EDS and depressive symptoms in patients with OSA.

## Introduction

Excessive daytime sleepiness (EDS) is an important public health concern and an independent predictor of increased health care use for outpatient physician visits and all-cause hospitalization (1). EDS has an important impact on general health and functional status, specifically influencing self-perceptions regarding energy/fatigue (2). EDS is one of the cardinal symptoms of obstructive sleep apnea (OSA), but its associations with severity of OSA have been inconsistent (3, 4), even in patients with severe OSA (5, 6).

Depression is one of the most common psychiatric illnesses in teenagers and adults and is a major public health problem, which is common in many different medical illnesses, including OSA with EDS (7). High degree of suspicion for OSA in patients with major depressive disorder (MDD) is warranted and for patients with treatment-resistant depression (TRD) (8). The prevalence of EDS in MDD is also greater than that reported in the general population (9). Patients with sleep apnea/hypopnea syndrome commonly demonstrate depressive symptoms and impaired daytime performance, yet it is unknown whether EDS is an actual clinical phenomenon or purely a result of overlapping psychological/somatic factors shared by both disorders. Its identification and characterization constitutes one of the most important challenges for sleep clinicians, not only for the diagnosis of OSA and depression but also for determining the response to treatment.

Our goal was to carry out a systematic review for exploring the prevalence of EDS and ascertaining the possible links between OSA, EDS and depression. At the same time, we want to analyze if continuous positive airway pressure (CPAP) is effective for improving the symptoms of EDS and depression in patients with OSA.

## Methods

We searched PubMed to identify papers published in English. We combined keywords and medical subject indices referring to terms related to EDS syndrome (Excessive daytime sleepiness OR daytime sleepiness), terms associated with sleep apnea syndrome (sleep apnea, obstructive sleep apnea, obstructive sleep apnea syndromes, sleep disordered breathing), and terms related to depression (depression OR mental disorder OR mood). Additionally, the selected papers had to meet these criteria for inclusion: (1) accessible in English; (2) human studies; (3) published between January 1990 to January 2021 unless related to some basic concept; and (4) abstracts were available. The criteria for exclusion were: (1) abstracts from conferences; (2) commentaries; (3) subjects aged <18 years; (4) reviews.

**Figure 1:**
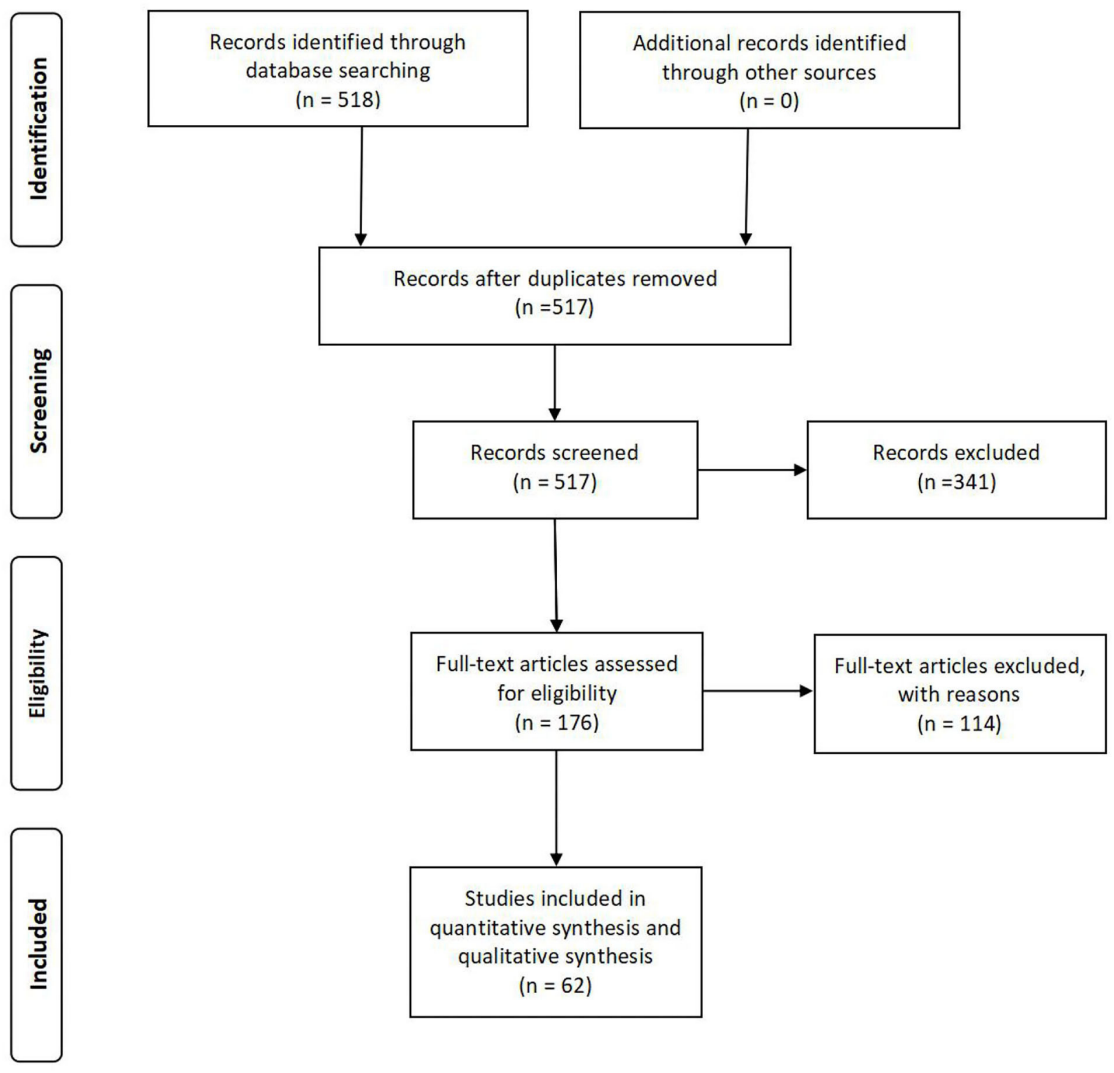
A prisma flow diagram for literature search.

The original search returned 518 articles. According to the inclusion and exclusion criteria, the abstracts were reviewed by a panel of three reviewers, and 176 full-text articles were retained for further analysis after removing duplicates and review articles. After a complete inspection of the full texts, 114 papers were excluded for different reasons. The current review focused on the remaining 62 articles (Figure 1).

Because of the narrative character of this review, we did not assess the risk of bias of the individual studies, nevertheless, all studies were scrutinized by three reviewers, who discussed the methodology and selected only studies according to the inclusion and exclusion criteria.

## Prevalence of EDS

### General Population

There is a lack of a standard definition of EDS, however, most studies use the Epworth Sleepiness Score (ESS) to assess sleepiness, which was quantified and dichotomized into presence (score >10) or absence (score ≤10) of EDS based on evidence indicating that individuals with scores >10 had an increased propensity to OSA syndrome, narcolepsy, or idiopathic hypersomnia.

EDS is common worldwide, and affects an estimated 9% of 16–84-year-old adults in New Zealand (10). In Australia, an estimated 12.6% of men have EDS (11). A cross-sectional multi-ethnic study estimated that 14.1% of the middle-to-older-age population had EDS (12). In China, 9.23% of women and 10.93% of men had complaints of EDS (13). In a prospective study, among the subjects without EDS at baseline, the 5-year incidence of EDS was 5.1%, but 43.8% of patients had persistent EDS 5 years later (14). EDS is more prevalent in individuals aged <30 years, indicating the presence of unmet sleep needs and depression, and older people aged >75 years, indicating increasing medical illness and health problems (4). Even in some large, relatively young and/or healthy samples, EDS was common.

### OSA

Sleep apnoea results from partial or total occlusion of the upper airway during sleep, causing hypopnea or apnea that leads to intermittent hypoxia, arousal from sleep, with resulting sleep fragmentation and disturbed sleep architecture. The most commonly encountered cause of EDS in a clinical setting is OSA. OSA can be reliably diagnosed using overnight polysomnography to assess the number of apneas–hypopneas per hour of sleep: the apnea hypopnea index (AHI).

Compared with the general population, the prevalence of EDS in patients with OSA is even higher, and has been noted in 39.5 (15) or 36.69% (13) of men and 21.18% (13) of women. Wahner-Roedler et al. found that more than half of all patients in both sexes reported symptoms of EDS (16). In a sleep-clinic-based sample of 915 patients, EDS was present in 38.8% (17). EDS was defined in broad terms, using either EDSQ positive (often or almost always “feel excessively sleepy during the day”) + or ESS >10, and 172 participants with mild OSA were analyzed. Omobomi et al. reported higher prevalence rates of subjective sleepiness of 74.4% (18). The current analyses and results from these studies, in spite of this, have emphasized that the absence of sleepiness does not exclude a diagnosis of mild OSA.

### Depression and Other Specific Population Subgroups

The prevalence of EDS in patients with depressive symptoms and insomnia is also greater than that reported in the general population. Data from 703 individuals with MDD were retrospectively collected from the sleep laboratory research database. The prevalence of EDS was 50.8% (9). Among 18 adolescents in Canada with TRD, 39% reported at least mild levels of EDS (ESS ≥10) (19). Among 1,311 people with insomnia, 45.61% reported EDS, which is a common complaint for individuals in this subpopulation (20). The reason for the high proportion of EDS, which has previously been described as a consequence of sleep-disordered breathing, may be that it is one of the overlapping symptoms of depression, insomnia, and OSA.

In other specific population subgroups, EDS has been reported in 19.1% (21) of commercial motor vehicle drivers, and in 14.9% (22) and 15.0% (23) of healthy elderly people. EDS has been reported in 18.9% (24) of patients with cardiovascular disease, 55.5% (25) of patients with type 2 diabetes, 14% (26) of patients with Fabry disease, 16.5% (27) of patients with sarcoidosis, and 17.5% (28) of patients with epilepsy. These data indicate that when diagnosing a case with a complaint of EDS, which can be independent manifestations of sleep disorders (29), OSA should not be considered the only cause.

In summary, EDS, is reported to be common in patients with physical disease and even more common in those with OSA and depressive symptoms. EDS is highly prevalent and has a significant impact on patients' general health and quality of life. Most of the clinical and research studies have used one single subjective self-completion questionnaire (ESS) to assess EDS. Findings were robust to the ESS cut point to define sleepiness, while it is well-known that EDS has multiple dimensions and can be quantified objectively by physiological measures, such as the multiple sleep latency test (MSLT) (30). Nevertheless, whether MSLT represents the gold standard to diagnose EDS remains unclear.

### Relationship of Daytime Sleepiness and Depression

#### General Population

There is a growing number of population-based strong and consistent relationships between depression and EDS, and vice versa (10–12). Alcántara et al. reported that although OSA (defined as AHI >15 or ≥30) was not associated with depression, sleep apneasyndrome (AHI >15 and EDS) was adversely associated with depression, it is possible that sleepiness might drive this association (12). It has been shown that depression is the most significant risk factor for EDS (4) and is associated independently with daytime sleepiness (31). These results suggest that a particular relationship exists between EDS and the severity of depression. Some studies have tested the hypothesis of whether daytime sleepiness largely contributes to the appearance of mood disorders, and vice versa. A multivariate logistic regression revealed that depressive symptoms were independent predictors of 5-year incidence of EDS, even after adjustment for some confounders (14). Analogously, EDS with or without OSA was associated with 7.5-year incidence of depression in both sexes, and the potential mechanisms may be physical inactivity, limited engagement in mastery activities, and sedentary lifestyle (32). In summary, as EDS may be an early sign of depression, it appears that it should be assessed and treated with cognitive-behavioral or pharmacological treatments to help prevent the onset of MDD episodes.

#### OSA

Prevalence studies have shown high rates of depression among patients with OSA in both community and clinical populations, and patients with OSA experience EDS and are more likely to have depressive symptoms (7), which are more common in women with OSA (33–35), and more severe than in men (35). Anti-depressant use is common in patients with OSA (36). For patients without an assessment for OSA but presenting with depression and sleepiness, the presence of OSA should be suspected (6).

Studies have shown conflicting results on the relationship of EDS and depressive symptoms with the severity of sleep apnea, respectively. Cumulative effects of undiagnosed and diagnosed sleep apnea, such as chronic sleep deprivation, cognitive/affective dysfunction, sleep disruption, and hypoxemia, likely contribute to psychological impairment (37). Unexpectedly, no significant correlation was seen between the severity of OSA and depression (5, 6, 33, 38), even after controlling for other factors (7). Although the presence of OSA can make an individual more likely to have depressive symptoms, there is another mediator, such as structural changes in sleep, rather than respiratory events between depressive symptoms and OSA (5).

Depression scores are positively correlated and significantly associated with sleepiness as measured by ESS in patients with mild (18), mild and moderate (38), mild to severe (33), moderate and severe (39), and severe (40) OSA, even after controlling for sex, body mass index (BMI) and AHI (39). Depressive symptoms are important contributing factors to EDS (17, 41), and a history of depression is a predictor of residual EDS in CPAP-treated OSAS (42). The severity of EDS is also the strongest predictor of depression in patients with mild to severe (32) and severe (33) OSA. The bidirectional relationship between depression and EDS also suggests that OSA patients with symptoms of EDS have the highest risk of associated depressive symptoms, and may benefit most from depression screening. EDS should be a target of our preventative strategies for depression.

It is, however, possible that ESS does not allow differentiation of depressive symptoms from real daytime sleepiness, and that objective sleepiness measurement, which was not performed in many studies, could provide different results. It may be that the different approaches and inappropriate psychological instruments, such as rating scale for depressive symptoms, with the absence of appropriate diagnosis based on DSM-IV or ICD-10 taxonomies in the diagnostic process that have led to confusion over the occurrence and nature of mental health difficulties in OSA.

#### Depression

EDS is a common symptom in individuals with MDD or severe depression and interventions (such as antidepressants or benzodiazepines) are possible for most risk factors of EDS (9). Few studies have investigated the relationship between EDS and depression in the subpopulation of individuals with MDD, and prospective studies should be conducted to confirm whether the presence of EDS is a marker of severity and which justifies particular care strategies for these individuals.

### EDS and OSA

#### General Population

EDS is considered to be the dominant sign of OSA. Around 3% of 1,011 Australian adults reported diagnosis of OSA and elevated ESS, and EDS (ESS ≥11) was associated with diagnosed and undiagnosed OSA, which was estimated using self-reported frequent loud snoring and witness apnea (3). Bixler et al. reported that in a random sample of 1,741 men and women there was only a weak association between AHI and EDS (4). More studies based on a large community-based sample are needed to determine whether EDS can be used as a diagnostic marker and considered an etiological factor for OSA screening in the general population.

#### OSA

The association of EDS and OSA severity is inconsistent. Some studies have shown that OSA severity (AHI) is associated with the frequency of sleepiness, and higher AHI is associated with more daytime sleepiness (5, 43, 44). By comparison of ESS scores among subgroups, Lee et al. showed that the severe OSA subgroup had a significantly higher ESS score than the mild and moderate subgroups had (5). Multiple linear regression analysis has shown that AHI is an independent factor influencing ESS score (45). These studies have suggested that subjective daytime sleepiness in patients with OSA syndrome is influenced by the severity of respiratory disorder indices.

Lee et al. developed the Subjective Apnea Severity Questionnaire to measure subjective OSA symptoms during the night and on waking in the morning, which were associated with daytime sleepiness in adults with moderate and severe OSA. However, they found that there was no significant relationship between all classes of OSA as defined by the AHI and EDS (46). The subjective and objective measures of OSA severity have different impacts on EDS. Analogously, AHI was not correlated with ESS in 49 newly diagnosed, untreated OSA patients without major comorbidities (6). These findings suggest that mechanisms other than the number and frequency of hypoxic events contribute to adverse effects of daytime sleepiness in these patient populations and explain to some extent why CPAP at times fails to improve EDS in patients with sleep apnea.

#### Depression

The rate of unsuspected and undetected OSA in 125 suicidal adults with MDD (8) and mood disorder patients (MDD or bipolar disorder) (47) was significant, but it is regrettable that the degree of daytime sleepiness did not predict AHI severity (8), which did not have a significant association with depression severity (47). We need to conduct investigations with greater numbers of patients with depression or comorbidity with OSA and verify whether improvements in depressive symptoms can be expected with the treatment of EDS and OSA.

### Effect of CPAP on EDS and Symptoms of Depression in OSA

Various conservative behavioral therapies are also used for OSA and CPAP is commonly chosen to treat OSA. Numerous studies have been conducted to evaluate the efficacy and/or effectiveness of CPAP in treating a wide range of OSA symptoms. Baseline sleepiness is the only factor predictive of compliance (48), and a high depression score is associated with non-compliance with CPAP (49). When nasal CPAP is titrated properly, hypopnea and apnea in patients with OSA syndrome are abolished completely, and arousal and oxygen desaturation disappear during CPAP once OSA is successfully treated (50). Since repetitive arousal or oxygen desaturation in brain tissue during sleep presumably contributes to the development of depression, a more complete understanding of the relationship between OSA and depression can be appreciated by examining changes in depressive symptoms (49, 51).

#### Short-Term (2–3 Months) Treatment

A beneficial effect of short-term (2–3 months) treatment following CPAP on EDS (48, 50, 52–58) and depression (37, 48, 50, 52, 55, 56, 58) has been seen in many studies. Both somatic and affective/cognitive symptoms on the Beck Depression Inventory (BDI) improved equally after 3 months' CPAP treatment for OSA (37). Treatment adherence to CPAP in the range of up to 4 h per night independently improved EDS, and adherence for 4–6 h per night independently improved all scales: ESS, Zung's Self-Rating Depression Scale (SDS), Fatigue Severity Scale (FSS), and Pittsburgh Sleep Quality Index (PSQI) (55). Persistent difficulties included lowered activity level and residual sleepiness in some individuals (57). Combined intervention with patient education and progressive muscle relaxation can significantly improve CPAP adherence in OSA patients for at least 12 weeks (59). In a randomized controlled crossover trial of CPAP, after 8 weeks of treatment, CPAP improved self-reported symptoms of daytime sleepiness more than did placebo but did not improve objective (MSLT) or subjective (ESS) measures of EDS and mood score (Profile of Moods States and BDI) (60). EDS and mood score do not always resolve even with optimal treatment with CPAP, suggesting that sleepiness and depressive symptoms may be related to other coexisting factors and comorbidities.

The reasons for these differences are not clear. First, there are large placebo effects for sleepiness and mood. Second, patients in these studies may have been more neurobehaviorally impaired for the similar levels of AHI severity. Third, CPAP use, the overall mild degree of neurobehavioral impairment in these patients, or morbidity not associated with OSA may be responsible for the failure to detect positive treatment effects of CPAP (60). Lastly, most previous studies evaluating the depressive symptoms in patients with OSA who were treated with CPAP included individuals with depression scores mainly in the non-clinical range, which may be related with floor effects of any intervention.

#### Long-Term Treatment

The results of most studies were based on a 12-week follow-up, which might limit the ability to generalize from these results to long-term effects of adherence on symptoms of depression and EDS. In a long-term prospective cohort study of CPAP treatment, adherence for 6 months (61) or 2 years (62) CPAP therapy for patients with severe OSA mitigated the impact of symptoms on work including EDS, impairment of workability, and depressive disorders. A multicenter, prospective cohort study including 300 patients with OSA and symptoms of depression prior to treatment demonstrated a significant improvement of depression score in response to CPAP. However, almost 42% of patients displayed persistent depressive symptoms after 1 year of CPAP use (51). Yamamoto et al. found that 2 years of CPAP treatment improved EDS and mood in 46 patients with severe OSA, and this effect may have contributed to an improvement of quality of life because most patients with severe OSA compromise their social activities (62).

These findings emphasize the importance of close follow-up of EDS and depressive symptoms once CPAP treatment is initiated in patients with OSA. It is regrettable that most of the current studies were related to OSA with depressive symptoms, and there is a lack of studies on CPAP for treatment of OSA comorbid depression. Skepticism has been expressed concerning the effectiveness of CPAP because of a lack of randomized controlled trials.

The non-specific effect of OSA on performance, mood, interest, sexual desire, and somatic state (each all approached in BDI) should be noted, which can be similar in other somatic conditions with pain, discomfort, and so on. Depressive symptoms can really improve if the underlying somatic condition does. It is an additional question if there is a biological link between OSA and mood disorder, probably as a confounding factor to the effectiveness of CPAP, but not possible to clarify by the present means.

## Conclusion

EDS is a widespread problem in the community with medical, societal and economic consequences, and is considered to be the major and most distinctive symptom of OSA. Similarly, MDD and many other diseases are comorbid with EDS. High prevalence rates of sleepiness in OSA patients have been reported; nevertheless, the association between EDS and AHI has been shown to be inconsistent. The relationship between depression and EDS is bidirectional, which supports the need to evaluate mental health whenever individuals in the general population present with EDS and OSA as a consequence, and comprehensive screening and testing for EDS should be considered in depression. More studies are needed to justify improved management of EDS and OSA to avoid negative consequences in patients with depression.

The underlying mechanism for EDS in patients with depression and OSA needs to be investigated further to make more specific therapeutic recommendations. It is pivotal to explore specific strategies targeting such factors and it will be interesting to reproduce these studies using an objective measure of EDS (such as MSLT) since few studies have used these methods and targeted patients with OSA and comorbid depression.

Effective therapies for patients with OSA not only improve OSA itself but also are likely to contribute to management of the comorbid conditions and their clinical consequences. The gold standard treatment is CPAP, which is effective in reducing AHI and improving objective and subjective measures of sleepiness and depressive symptoms (Table 1). A minimal therapeutic duration of 4 weeks is needed if CPAP is used to treat patients with OSA for associated EDS impairments or deficits. Numerous studies of CPAP have also highlighted the importance of assessment and intervention targeting psychosocial functioning and sleepiness in individuals with OSA before and after treatment.

**Table 1:** CPAP and its therapeutic effects on EDS and depression.

**Type of study**	**OSA severity**	**Criteria used to define depression**	**OSA+CPAP (*n*)**	**Controls (*n*)**	**CPAP duration**	**Therapeutic effects of CPAP**	**References**
Cohort study	AHI unclear	Beck Depression Inventory (BDI)	39	—	3 months	Improved BDI scores	(37)
CT	AHI>20	Zung's Self-Rating Depression Scale(SDS)	132	38	2 months	Improved SDS and ESS scores	(50)
CT	Moderate to severe	BDI	37	27	3 months	Improved BDI and ESS scores	(57)
Cohort study	Moderate to severe	BDI-II	16	—	3 months	Improved ESS but no effect on BDI-II	(54)
Cohort study	AHI ≥15 or <15 with complications	Zung's SDS	76	—	3 months	Adherence at 4–6 h per night improved SDS and ESS scores	(55)
Cohort study	AHI >10	Hospital anxiety-depression scale (HAD)	36	—	2 months	Improved HAD and ESS scores	(48)
CT	AHI ≥15	HAD	21	16	3 months	Adherence>4.5 h per night improved MSLT and HAD	(56)
RCT	Women with AHI ≥15	HAD	151	156	3 months	Improved HAD and ESS scores	(52)
Cohort study	Snoring, EDS and AHI >5	—	17	—	3 months	Improved ESS scores	(53)
Cohort study	AHI ≥15	BDI-II	43	—	3 months	Improved BDI-II and ESS scores	(58)
RCT	5<AHI <30	Profile of Moods States (POMS), BDI	28	4	3 months	Did not improve MSLT, ESS, POMS, and BDI	(60)
Cohort study	AHI ≥30	HAD	40	—	6 months	Improved HAD and ESS scores	(61)
Cohort study	Male diagnosed with severe OSAS	SDS	46	—	2 years	Improved HAD and ESS scores	(62)
Cohort study	QD2A score ≥7	13-item, self-rated Pichot depression scale (QD2A)	300	—	Average of 529 days	Improved QD2A scores but 41.7% presented persistent depressive symptoms	(51)

*AHI, apnea/hypopnea index; RCT, randomized controlled trial; CT, controlled trial; OSA, obstructive sleep apnea; ESS, epworth sleepiness score; HAD, hospital anxiety-depression scale; MSLT, multiple sleep latency test*.

## Data Availability Statement

The original contributions presented in the study are included in the article/supplementary material, further inquiries can be directed to the corresponding authors.

## Author Contributions

DZ contributed to conception and design of the paper, searched PubMed to identify studies and wrote the first draft of the manuscript. ZZ, HL, and KD scrutinized all studies and wrote sections of the manuscript. All authors contributed to manuscript revision, read, and approved the submitted version.

## Conflict of Interest

The authors declare that the research was conducted in the absence of any commercial or financial relationships that could be construed as a potential conflict of interest.

## Publisher's Note

All claims expressed in this article are solely those of the authors and do not necessarily represent those of their affiliated organizations, or those of the publisher, the editors and the reviewers. Any product that may be evaluated in this article, or claim that may be made by its manufacturer, is not guaranteed or endorsed by the publisher.
